# PICKLES: the database of pooled *in-vitro* CRISPR knockout library essentiality screens

**DOI:** 10.1093/nar/gkx993

**Published:** 2017-10-25

**Authors:** Walter F Lenoir, Tassica L Lim, Traver Hart

**Affiliations:** Department of Bioinformatics and Computational Biology, The University of Texas MD Anderson Cancer Center, Houston, TX, USA; UTHealth Graduate School of Biomedical Sciences, The University of Texas MD Anderson Cancer Center, Houston, TX, USA

## Abstract

The adaptation of CRISPR/Cas9 systems for pooled library genetic knockout screens in mammalian cells has substantially advanced the state of the art in human functional genomics. Screening panels of cell lines for genes whose knockout imposes a significant fitness defect has dramatically expanded our catalog of high-confidence essential genes, and has already proven useful in identifying tumor-specific essential genes for the development of targeted therapies. However, nonexperts currently lack an easy to use way to access this data and to identify whether their genes of interest are essential across different genetic backgrounds. The volume of screening data is expected to grow massively, making the problem more intractable. Here we describe PICKLES, the database of Pooled *In vitro* CRISPR Knockout Library Essentiality Screens, where end users can display and download raw or normalized essentiality profiles for more that 18 000 protein-coding genes across more than 50 cell lines. An additional data set with 15,000 genes targeted by pooled library shRNA in over 100 cell lines is also included. Researchers can see at a glance the relative fitness defect and tissue specificity of their genes of interest, generate and save figures locally, and download all raw data. The database is available at http://pickles.hart-lab.org.

## INTRODUCTION

The ability to knock out a gene and observe the resulting phenotype has been a foundational tool for functional genomics for decades. The yeast deletion library has been extensively studied, and recently a near-complete catalog of fitness defects of all pairwise deletions of yeast genes was published. The tractability of yeast genetics made Saccharomyces cerevisiae a powerful model system. The discovery of RNA interference and its adaptation to RNA-guided transcript knockdown brought large-scale genetic screens to higher eukaryotes (1,2) but imprecise targeting, low penetrance, and off-target effects (3–5) led to a loss of confidence in this method for large-scale screens (6). Recently, the application of CRISPR/Cas9 technology to generate double strand breaks in target DNA, whose repair by nonhomologous end joining frequently results in indels, has been exploited to knock out protein coding genes in a variety of model systems by targeted introduction of frameshifts or other deleterious mutations (7,8).

Genome-scale CRISPR libraries have been adapted to a variety of screening goals, including knockout libraries for loss of function screens for protein coding genes (9) (10) and noncoding RNA (11,12). The most commonly used CRISPR-associated endonuclease, SpCas9, has been modified to disable its endonuclease activity, facilitating protein fusion with domains for transcriptional activation (13,14), transcriptional repression (13,15), and chromatin modification (16). Multiplexed guide designs have been engineered to enable pairwise gene perturbation screens to detect synthetic lethal genetic interactions (17) and to remove precisely targeted segments of DNA (12).

Despite this breadth of available technologies, the most common application of pooled CRISPR libraries is to screen protein coding genes for knockout fitness defects in cancer and other human cell lines. Pooled library screens in cancer are designed to identify the essential genes specific to tumors of a given tissue of origin or even subtype. Early screens demonstrated the power of this differential essentiality approach (18,19) and demonstrated that genotype-specific vulnerabilities could be identified and targeted (20), while subsequent efforts expanded the scope of the cell lines being screened (21,22), and vastly more data is in the pipeline (23,24) (Meyers *et al.*, bioRxiv, 2017).

As this massive screening effort expands, so grows the need for a central repository where researchers and the public can easily interpret the data. Here we present PICKLES, the database of Pooled *In vitro* CRISPR Knockout Library Essentiality Screens. PICKLES presents a easy to use interface where a user can visualize how the essentiality of a given gene varies across experiments and across tissues/cells probed within an experiment. Raw data from large-scale screening efforts is processed through the BAGEL pipeline (25), which generates a log Bayes Factor that represents the confidence level of whether a gene is essential in a given cell line screen. Both raw and normalized BFs are available for download.

The PICKLES database currently contains data from four unique CRISPR knockout libraries applied in screens of over 60 cell lines, performed in at least six labs. It additionally contains data from genome-scale shRNA knockdown screens in over 100 cancer cell lines (26–28). We anticipate expanding this database as additional large scale screening data are made available.

## DATA SOURCES AND PREPROCESSING WITH BAGEL

Viral-mediated, pooled library CRISPR screens involve transducing a large population of cells with a pooled library of CRISPR reagents (guide RNAs, or gRNA). Expression of SpCas9 or a related endonuclease, either from prior genetic knock-in or encoded on the same viral backbone as the gRNA, results in gRNA-mediated cleavage and, in most cases, error-prone repair of targeted loci. Successful targeting of a fitness gene results in mutation or indels resulting in frameshift, loss of gene function, and subsequent cell death, arrest, or severe fitness defect, causing cells harboring that gRNA to represent an ever smaller fraction of total transduced cells as generations pass. At an endpoint, typically 8–15 doublings after library transduction, gRNA sequences are amplified from genomic DNA and sequenced and their relative abundance is compared to either a control timepoint immediately after infection or to the original plasmid pool. Guide RNA targeting essential genes will be depleted in the final pool, resulting in a strong negative fold change relative to genes with no fitness defect.

**Figure 1.**
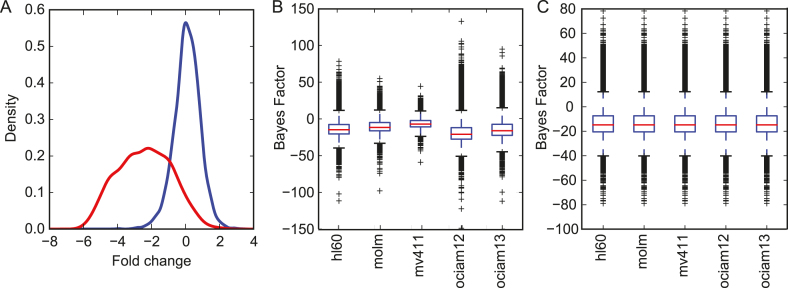
(**A**) Density plot of fold changes of gRNA targeting essential (red) or nonessential (blue) genes. (**B**) Distributions of BAGEL Bayes Factor (BF) scores in Tzelepis AML dataset. Cells are screened under uniform conditions but experimental and biological differences drive variance in results. (**C**) Quantile normalized BFs of the Tzelepis AML dataset, allowing for direct gene BF comparisons across cell lines.

Raw read count data from all datasets was acquired and processed with BAGEL (25). BAGEL is a Bayesian classifier trained using gold standard reference sets of essential and nonessential genes. The observed fold changes of gRNA targeting uncharacterized genes are compared to the observed fold change distributions of gRNA targeting genes in the training sets and a log Bayes Factor (BF) is calculated. The BF represents the relative confidence that the gene is essential (i.e. that the observed fold changes were more likely drawn from the essential or nonessential distributions; Figure 1A).

The BF for a given gene in a given screen is a function of the number of gRNA targeting that gene, the number of replicates screened, and the number of doublings at the screen endpoint, as well as other global experimental factors. It is also common for BF distributions to vary considerably even within the same set of experiments/screens conducted in a single lab (Figure 1B). For this reason, we have quantile normalized the BF from each experimental set before generating the display (Figure 1C). Both raw and quantile normalized BF data are available for download from the website. However, across-dataset comparisons are still problematic. The different CRISPR libraries generally have different numbers gRNA per gene, as well as wide variation in gRNA knockout efficiency, and the various screening groups often implement experimental designs that differ in number of replicates and timepoints assayed. The BAGEL pipeline accumulates information from each gRNA in each replicate at each timepoint, leading to highly accurate results within a dataset but strong batch effects between datasets. We therefore display the results from each dataset independently.

### shRNA data

In addition to CRISPR data, the PICKLES database also contains a large compendium of pooled library shRNA screen data from (26–28). This data has undergone considerably more thorough preprocessing and filtering, including reducing the number of screens to 112 high quality screens, ensuring minimal representation of shRNA in T0 populations, and normalizing to the number of shRNA targeting each gene in each experiment, in order to yield a robust dataset with minimal false positives. These data processing steps are described in (Hart *et al.*, bioRxiv, 2017). Table 1 shows a complete listing of the data available at time of writing. We note that, in general, CRISPR screens show much greater sensitivity and specificity than shRNA screens (29) and that global analyses such as those presented here are less affected by these QC considerations.

**Table 1.** Fitness screens currently available in PICKLES

Screen/library	Data type	Number of genes	Number of cell lines
shRNA	Essentiality Score	13 395	112
GeCKO	Quantile Normalized Bayes Factor	15 466	33
TKOv1	Quantile Normalized Bayes Factor	17 230	10
Tzelepis/Yusa	Quantile Normalized Bayes Factor	17 997	5
Wang	Quantile Normalized Bayes Factor	19 161	19

## DATABASE INTERFACE AND TUTORIAL

**Figure 2.**
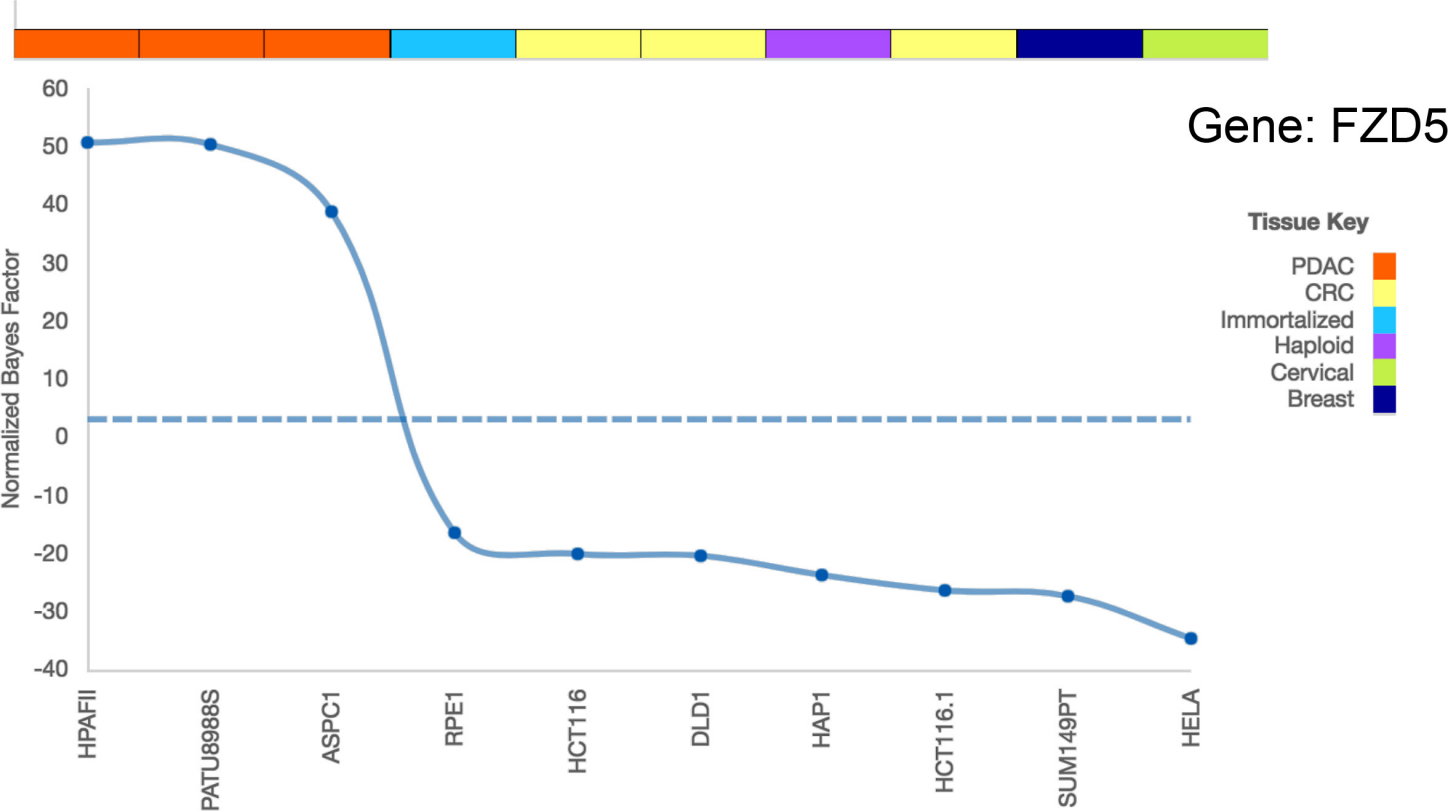
BFs of FZD5 in the TKOv1 library dataset. Dashed blue line indicates a threshold for gene essentiality (BF = 3). The tissue key displays the cell line tissue/tumor subtype of origin. The FZD5 receptor is essential in PDAC cells (orange), with all PDAC Bayes Factors falling well above the indicated threshold.

The PICKLES database can be found at pickles.hart-lab.org. The main database display tool is found under the ‘Essentiality Map’ tab. Upon entering a valid gene symbol in the input form, the essentiality profile for that gene will be plotted for each data set where that gene is assayed (30). For each display, the primary y-axis plots the gene BFs (blue dots connected by a line), as well as a dashed line at BF = 3 (dashed blue) representing a low-stringency threshold for gene essentiality (29) (Figure 2). Above the plot is a color-coded bar representing the cancer subtype or tissue of origin for the cell line; the key is to the right of the plot. Figure 2 shows the essentiality plot for the FZD5 receptor, which is specifically essential in RNF43-mutant pancreatic ductal adenocarcinoma (PDAC) cells (20). As with all essentiality plots, the figure can be saved locally in png format and the data can be downloaded in a tab-delimited text file.

**Figure 3.**
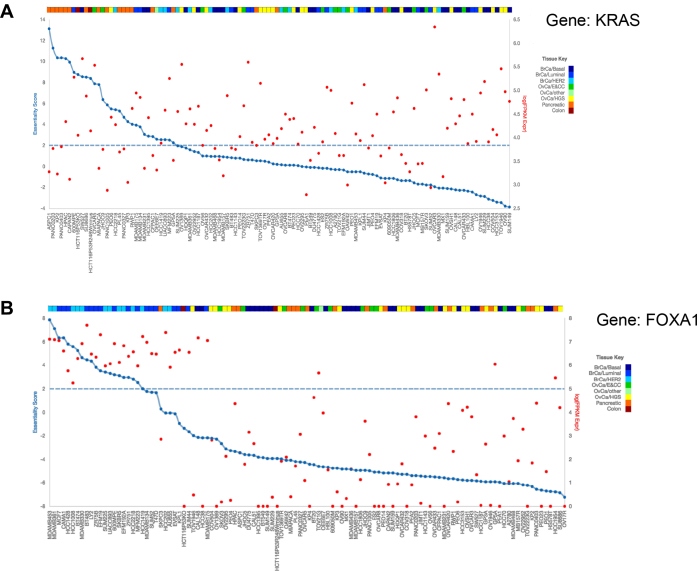
(**A**) BFs of KRAS in the Achilles library dataset (blue), with gene expression data (red). Pancreatic (tissue key; orange) and specific lung (tissue key; cyan) cancer cell lines have high BFs consistent with KRAS dependence in KRAS-mutant cancers. (**B**) BFs of FOXA1 using the shRNA library dataset. Both gene expression (red) and BFs (blue) are high in luminal and HER2 breast cancer cell lines compared to basal breast, ovarian, pancreatic and colon cancer cell lines.

Where available, the target gene expression level is displayed on the same graph. For example, Figure 3A shows the essentiality plot for KRAS in the Project Achilles screens (22), and demonstrates the tissue-specific dependence on KRAS of PDAC and select lung cancer cell lines. Expression level of KRAS drawn from CCLE microarray data is plotted for each cell line in red (right Y axis). In this case, no obvious correlation between expression and essentiality exists. In contrast, breast cancer oncogene FOXA1 (Figure 3B) shows high essentiality and high expression only in HER2+ and luminal breast cancer cell lines.

Other tabs on the top navigation bar provide links to standard resources. The statistics tab shows summary statistics of the database; at time of writing, these data are summarized in Table 1. Raw data and processed (quantile normalized) data for all screens can be downloaded from the Documents tab, as well as links to the published studies from which these data are derived.

## IMPLEMENTATION AND FUTURE DIRECTIONS

The data display is currently implemented exclusively in javascript, using the charts.js library for display. A sqlite database containing all gene essentiality and gene expression data resides on the web server and is queried through a custom python web service. The browser-based javascript modifies the view in response to user selection of options and downloads additional data via http request when the user searches for a new gene. Currently statistical tests are pre-calculated for the existing data and are loaded as static metadata.

## CONCLUSIONS

We present PICKLES, the database of Pooled *In vitro* CRISPR Knockout Library Essentiality Screens, where researchers can explore the gene essentiality profiles of their favorite genes across a large set of CRISPR knockout and shRNA knockdown fitness screens, mostly in cancer cell lines. Raw data from five major data sets of genome-scale screens, for a total of over sixty CRISPR-screened cell lines and over one hundred shRNA-screened cell lines, was acquired and processed with the BAGEL algorithm, resulting in a consistent set of essentiality scores. An easy to use interface allows users to visualize how gene-specific essentiality varies across tissue types and, in many cases, the relationship with gene expression levels in the same cells. We anticipate that this database will grow rapidly as hundreds of screens are known to be in the pipeline in screening labs around the world, and we envision that the PICKLES database will be a broadly useful tool for mining this important resource.
